# Spermatogenesis improved by suppressing the high level of endogenous gonadotropins in idiopathic non-obstructive azoospermia: a case control pilot study

**DOI:** 10.1186/s12958-018-0401-7

**Published:** 2018-09-22

**Authors:** Xuechun Hu, Zheng Ding, Zhiwei Hong, Zhichuan Zou, Yuming Feng, Ruilou Zhu, Jinzhao Ma, Xie Ge, Chaojun Li, Bing Yao

**Affiliations:** 1 Center of Reproductive Medicine, Nanjing Jinling Hospital, the Medical School of Nanjing University, Nanjing, 210002 China; 2 Nanjing Jiangning Hospital, the Affiliated Jiangning Hospital of Nanjing Medical University, Nanjing, 210000 China; 3 Department of Urology, Fujian Provincial Hospital, Fuzhou, 350000 China; 4 MOE Key Laboratory of Model Animals for Disease Study, Model Animal Research Center and the Medical School of Nanjing University, National Resource Center for Mutant Mice, Nanjing, 210061 China

**Keywords:** Gonadotropin reset, NOA, GnRHα

## Abstract

**Background:**

Elevated plasma gonadotropins were associated with desensitization of Sertoli and Leydig cells in the male testis. Testis spermatogenesis ability would be improved via inhibiting high endogenous gonadotropin in patients with severe oligozoospermia. Whether it would be beneficial for non-obstructive azoospermia (NOA) patients was still unclear.

**Methods:**

Goserelin, a gonadotropin releasing hormone agonist (GnRHα) was used to suppress endogenous gonadotropin levels (gonadotropin reset) in the NOA patients, improving the sensitization of the Sertoli and Leydig cells. Then human menopausal gonadotropin (hMG) and human chorionic gonadotropin (hCG) were injected to stimulate them to ameliorate the ability of testicular spermatogenesis. The main outcome measure was the existence of spermatozoa in the semen or by testicular sperm extraction (TESE). Elevation of inhibin B and/or ameliorative expression pattern of ZO-1 was the secondary objective.

**Results:**

A total of 35 NOA men who failed to retrieve sperm via TESE were enrolled. Among these, 10 patients without treatment were selected as control group and secondary TESE was performed 6 months later. Of the 25 treated men, inhibin B was elevated in 11 patients in the first 4 weeks (Response group), while only 5 patients had constant increase in the following 20 weeks (Response group 2). Of the 5 men, 2 men acquired sperm (Response group 2B), while 3 failed (Response group 2A). Immunofluorescence of mouse vasa homologue (MVH) and ZO-1 showed that both positive MVH signals and ZO-1 expression were significantly increased in the Response group 2, but only Response group 2B showed ameliorative ZO-1 distribution.

**Conclusions:**

Gonadotropin reset, a new therapeutic protocol with GnRHα, was able to improve the ability of testicular spermatogenesis in the NOA patients through restoring the sensitivity of Sertoli and Leydig cells, which were reflected by elevated inhibin B and ameliorative ZO-1 expression and distribution.

**Trial registration:**

ClinicalTrials.gov identifier: NCT02544191.

**Electronic supplementary material:**

The online version of this article (10.1186/s12958-018-0401-7) contains supplementary material, which is available to authorized users.

## Background

Non-obstructive azoospermia (NOA) affects approximately 1% of the general population and 10–20% of infertile men worldwide [1, 2]. A series of factors were associated with NOA, for example, hypogonadotropic hypogonadism (HH), Y microdeletion, chromosomal abnormalities etc. [3]. The causes and the underlying mechanism of idiopathic NOA still remain unclear. Testicular sperm extraction (TESE) or microdissection testicular sperm extraction (micro-TESE) combined with intracytoplasmic sperm injection (ICSI) was the approach recommended for idiopathic NOA [4]. However, the total rate of sperm retrieval was only about 50% [5]. Thus, efficient medical treatment strategies are required.

Hormone replacement therapy would improve the ability of the testis to produce spermatozoa in idiopathic NOA patients [6, 7]. For example, the improvement of spermatogonial DNA synthesis was demonstrated by Shinjo and coworkers [8], the elevation of intra-testicular testosterone levels was demonstrated by Kato and coworkers and the hypertrophic change of leydig cells was demonstrated by Oka and coworkers, respectively [9, 10]. Recently, a multi-institutional prospective study conducted by Shiraishi and coworkers provided a stronger evidence of the efficiency of hormone therapy [6]. However, the total rate of acquiring sperm was only about 10–20%. A possible explanation of the low success rate was that high plasma gonadotropins in the patients led to dysregulated function of FSH and LH receptors (FSHR, LHR) in Sertoli and leydig cells [7, 11, 12]. As demonstrated by in vivo and in vitro studies, desensitization and downregulation of FSH signaling in Sertoli cells was induced by the chronic stimulation of FSH [13–15]. Considering the risk of high plasma gonadotropins, a ‘gonadotropin reset’ with leuprolide acetate, a gonadotropin releasing hormone agonist (GnRHα), was proposed to induce a hypogonadotrophic state by Foresta and coworkers [11]. Thus, the FSHR and LHR in the testis would be ‘released’ and subsequent exogenous hormone stimulation would be beneficial for testis spermatogenesis, as great success has been achieved in the treatment of hypogonadotropic hypogonadism via hormone replacement therapy. Moreover, gonadotropin reset with GnRHα had been demonstrated to improve the function of Sertoli cells and subsequently enhance the sperm concentration in patients with severe oligozoospermia [11, 16]. However, to our knowledge, there is no data of gonadotropin reset with GnRHα in the NOA patients.

Inhibin B is secreted by Sertoli cells and is involved in the negative feedback of plasma FSH [17]. The expression of inhibin B was regulated by FSH and plasma inhibin B level was considered as a marker of Sertoli cell function [18]. Plasma inhibin B level was also closely related with spermatogenesis. Low levels of inhibin B was demonstrated in patients with bad semen quality which may be related with the dysfunction of Sertoli cells [11, 18]. Moreover, elevated inhibin B levels may indicate improved function of Sertoli cells, reflecting better spermatogenesis environment [11] .

Cell-cell junction in the seminiferous epithelium played an important role in spermatogenesis including self-renewability and differentiation of spermatogonial stem cells into mature spermatozoa [19]. Blood testis barrier (BTB) mainly includes tight junctions (TJ) that are present between adjacent Sertoli cells [19]. Redistribution of the TJs to the cytoplasmic compartment or decreased expression was associated with abnormal spermatogenesis [20, 21]. NOA patients were also accompanied with dysfunction of TJ proteins, such as occludin 11 etc. [21]. Zonula occludens-1 (ZO-1) is a membrane protein that distributed peripherally, and interacted together and anchored membrane proteins to the actin cytoskeleton [22]. However, the expression pattern of ZO-1 has never been reported in the NOA patients.

Hence, in the present study, we tried to suppress the high endogenous gonadotropin levels in the NOA patients with goserelin, another GnRHα to release and restore the receptors’ function and then stimulate them using human menopausal gonadotropin (hMG) and human chorionic gonadotropin (hCG) to improve the ability of testicular spermatogenesis. Inhibin B, ZO-1 and mouse vasa homologue (MVH, a marker of germ cells) were detected to evaluate the response to the intervention.

## Methods

### Subjects

**Fig. 1:**
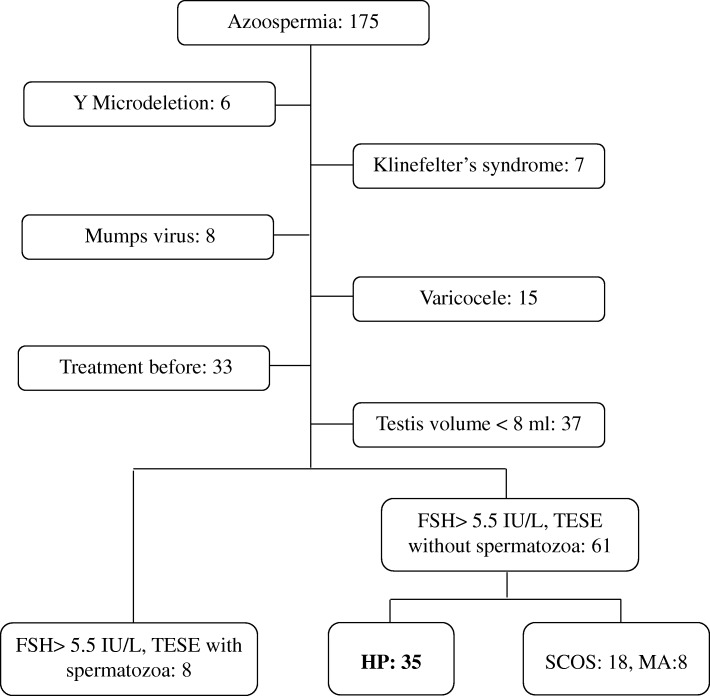
Patients selection process. A total of 175 patients with azoopspermia in the male infertility clinic were enrolled. Among them, 13 with genetic abnormality, 8 with mumps virus infection history, 13 with varicocele, 33 who received medical treatment, 37 with small testes, 8 with acquired sperm, 8 with MA and 18 with SCOS were excluded. The rest 35 patients diagnosed with hypospermatogenesis (HP) were enrolled

**Fig. 2:**
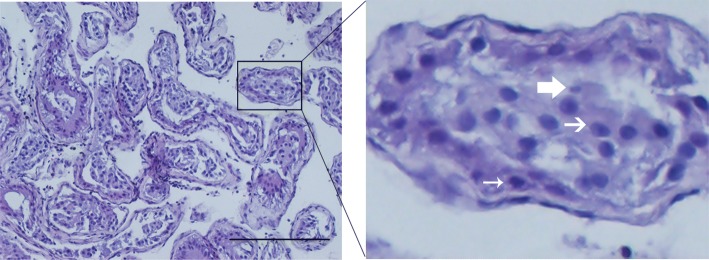
Histological analysis. The typical hematoxylin and eosin (H&E) staining of the testis specimens of the enrolled patients showed hypospermatogenesis (HP). The narrow arrow indicated the spermatocyte, the broad arrow indicated the elongated spermatid, and the medium arrow indicated the round spermatid. Scale bar, 200 μm

The study protocol was approved by the Research Ethics Committee of Nanjing Jinling Hospital and informed consent was obtained from all the participants. Semen samples from the patients with azoospermia were analyzed at least twice at an interval of 3 weeks according to the WHO Laboratory Manual for the Examination and Processing of Human Semen (5th edition) [23]. Totally, 175 patients aged between 18 and 45 with FSH plasma level > 5.5 IU/L were included. Patients with history of cryptorchidism (0), varicocele (15) or testicular trauma (0), medical treatment before (33), genital infections (8), Y Microdeletion (6), chromosomal abnormalities (7), both sides of the testes were less than 8 ml (37) and men who acquired sperm with TESE (8) were excluded (Fig. 1). Patients with Sertoli cell only syndrome (SCOS, 18) and maturation arrest (MA, 8) were also excluded. Finally, 35 patients diagnosed with hypospermatogenesis (HP) as shown by histological analysis in Fig. 2 were enrolled.

### Study design

**Fig. 3:**
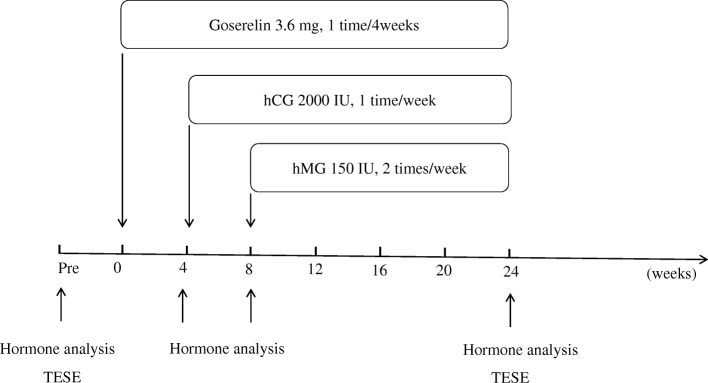
Study protocol. Patients were given goserelin once every 4 weeks for 24 weeks. hCG was administrated with a dose of 2000 IU for once a week for 20 weeks. hMG was injected at a dose of 150 IU for twice a week for 16 weeks. Twenty four weeks later, all patients received the secondary TESE. Plasma hormone analysis was performed at the first TESE, week 4, week 8 and week 24 of the treatment

The overall experimental design was presented in Fig. 3. 3.6 mg goserelin (AstraZeneca, UK Limited) was given to patients once every 4 weeks through subcutaneous injection for 24 weeks. hCG (Pregnyl, N.V. Organon Oss, Holland) was administered via intramuscular injection with a dose of 2000 IU, once a week for 20 weeks. hMG (Urofollitropin for Injection, Livzon Pharm Group Inc., China) was also administered through intramuscular injection at a dose of 150 IU, twice a week for 16 weeks. Ten patients who did not agree with the treatment were selected as the control group. All patients received the secondary TESE 24 weeks later.

### Clinical monitoring

All enrolled patients were informed of the possible side effects during the period of treatment. Informed consents were acquired from all the men. Semen parameters were analyzed by the computer aided semen analysis (CASA) system (WLJY-9000). Body mass index and testicular volume measurement were carried out. Testicular volume uses an ellipsoid approximation (volume = length * width * depth * 0.523) by ultrasound (nemio-XG580, Toshiba, Tokyo, Japan). Blood samples were obtained at 8–11 a.m. and centrifuged at 1800 g for 10 min. Plasma inhibin B was detected by electrochemical luminescence method (Roche, Mannheim, Germany). The levels of FSH, luteinizing hormone (LH), total testosterone, estrogen (E2) and prolactin (PRL) were determined by radioimmunoassay (Beckman Coulter, Brea, USA). The normal range of FSH is 1–5.5 U/L, 1–6.3 U/L for LH, 9.4–37 nmol/L for testosterone and 18.22–311.27 pg/mL for inhibin B. Apart from these, blood pressure, hematological parameters including blood corpuscle, biochemical and lipid parameters were detected to evaluate the safety of therapeutic protocol.

### H& E and immunofluorescence staining

Testes specimens were collected by testicular fine needle aspiration cytology (FNAC) for histopathological and immunofluorescent analysis as described previously [24]. Briefly, the testes were fixed in 4% paraformaldehyde. Following routine pathological procedure, the section slides were prepared. Subsequently, the slides were stained with hematoxylene-eosin (H&E). The steps required for staining of the testicular sections with ZO-1 (ab96587, Abcam, USA) and MVH (ab13840, Abcam, USA) antibodies were the same as described previously [25]. Briefly, the sections were hydrated with gradient alcohol followed by dewaxing with xylene, blocked with 5% bovine serum albumin (BSA, Sigma) for 1 h at room temperature and finally incubated with ZO-1 and MVH antibodies at 4 °C overnight. The following day, the samples were washed with PBS for three times, and incubated with anti-rabbit IgG H&L secondary antibody for 1 h in the dark. Finally, the sections were visualized using a fluorescence microscope. Quantification of MVH-positive signals and mean fluorescence intensity of ZO-1 per tubule area were calculated referring to the study of bai and coworkers [26] and then the data in NOA groups were normalized by OA group, 30 tubules from each group were calculated.

### Statistical analysis

All data were evaluated for normal distribution by the Kolmogorov–Smirnov test. The variables conformed to normal distribution was summarized as mean ± standard deviation, and those departed from normal distribution were summarized as medians and interquartile intervals. Differences between the groups at baseline and differences at different time points for each group were calculated by Student’s t-test. *p* < 0.05 was considered as statistically significant.

## Results

### Characteristics of studied men

The baseline data of all the participants were presented in Table 1. The plasma FSH and LH levels were significantly higher than normal range while the inhibin B was lower than normal range. The plasma total testosterone level and body mass index (BMI) were in the normal range. No parameters showed difference between the control group and the treatment group at the baseline.

**Table 1:** Patients’ characteristics of all the enrolled patients

Variables	Treatment	Control	*P*
Age	25.8 ± 3.4	26.6 ± 3.3	0.53
BMI (kg/m^2^)	22.2 ± 1.9	22.5 ± 1.1	0.70
FSH (IU/L)	18.8 ± 7.2	20.8 ± 5.6	0.43
LH (IU/L)	9.1 ± 3.6	10.2 ± 3.3	0.52
Total testosterone (nmol/L)	13.2 ± 4.3	12.6 ± 4.0	0.46
Estradiol (pmol/L)	127.9 ± 69.6	122.9 ± 47.2	0.84
PRL (mIU/L)	220.9 ± 81.1	203.7 ± 61.9	0.55
Inhibin B (pg/mL)	13.6 ± 13.1	14.1 ± 14.1	0.95
Testicular volume (mL)	10.7 ± 1.8	11.2 ± 1.9	0.46

### Therapeutic response of the plasma hormones

**Fig. 4:**
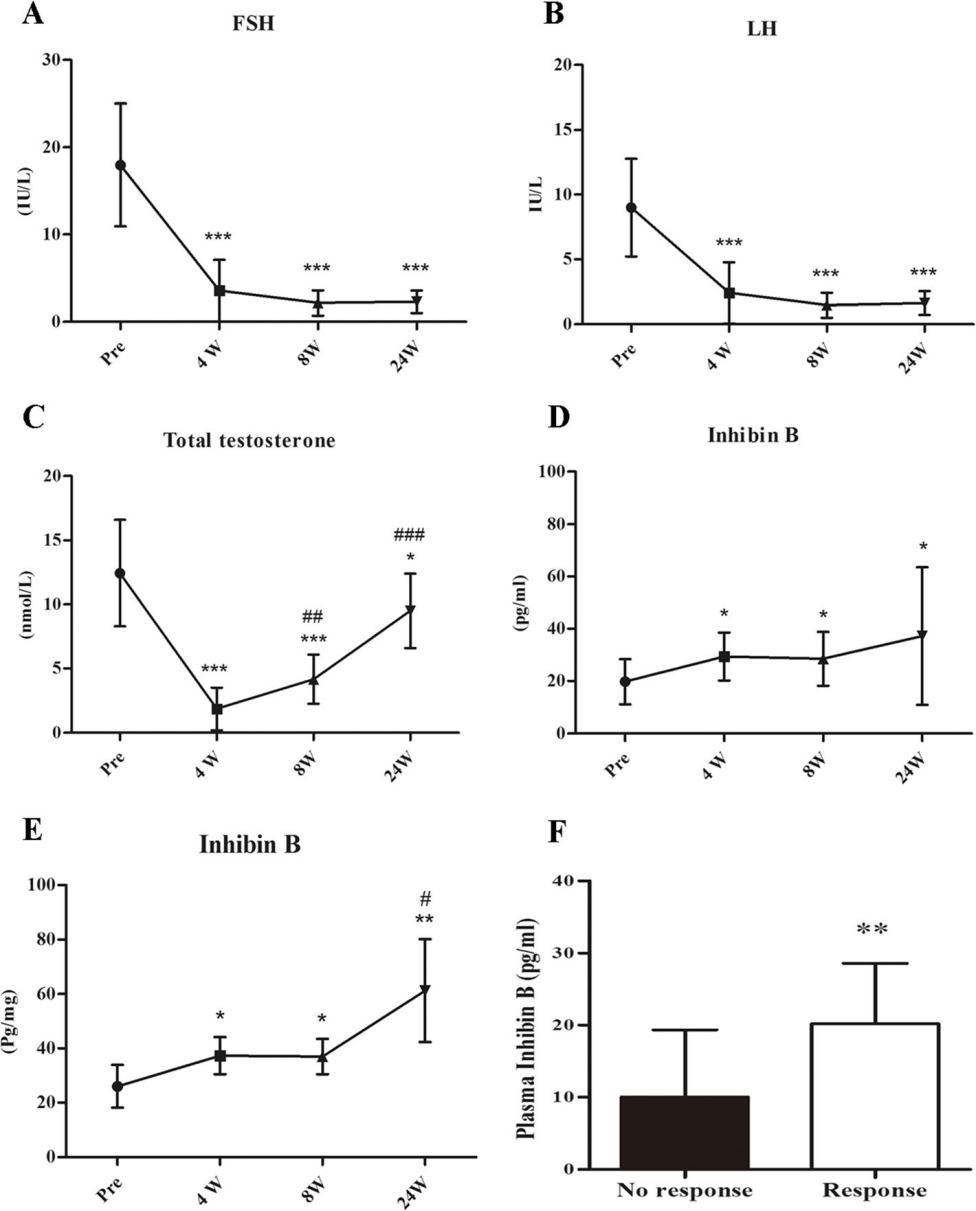
Dynamic changes in plasma hormone levels. **a**-**c** The dynamic changes of FSH, LH, T levels through the whole treatment period were shown. **d** The dynamic change of inhibin B in the Response group (Response group 1 + 2). **e** The dynamic change of inhibin B in the Response group 2. **f** The level of inhibin B at baseline in the No response group and the Response group (Response group 1 + 2). Results were shown as mean ± SD. **a**-**d** *P < 0.05 compared with the value before the treatment (Pre); ^**^P < 0.01 compared to the value before the treatment; ^***^P < 0.001 compared with the value before the treatment; ^##^*P* < 0.01 compared with value at week 4. ^###^*P* < 0.001 compared with value at week 4; **e** *P < 0.05 compared with the value before the treatment, ^**^P < 0.01 compared to the value before the treatment, ^#^*P* < 0.015 compared with value at week 8; f^*^P < 0.05 compared with No response group

The dynamic change of plasma hormones were shown in Fig. 4. The levels of plasma FSH, LH, and total testosterone were significantly suppressed by goserelin in the first 4 weeks (Fig. 4a-c). Later, hCG and hMG demonstrated little influence on the plasma FSH and LH levels, but total testosterone showed significant increase (Fig. 4c). Interestingly, 11 of the 25 treated patients showed elevated inhibin B levels with goserelin alone in the first 4 weeks and were considered as Response group (Fig. 4d), while the other 14 showed no change were seen as No response group. The baseline levels of plasma inhibin B were significantly higher in the Response group by further analysis (Fig. 4f). Among the 11 patients, 5 showed constant increase of inhibin B in the following 20 weeks (Response group 2, Fig. 4e), while the other 6 were hewed back to the baseline (Response group 1). Finally, 2 men in the Response group 2 acquired spermatozoa (Response group 2B), while the other 3 failed to acquire (Response group 2A). One man in the Response group 2B acquired sperm in the semen. Sperm concentration was 1.42 * 10^6^/ml and the total sperm count was 3.98*10^6^. No significant difference of PRL or E2 was observed during the whole process (data not shown). No sperm was found in the secondary TESE in the control group and no significant change of plasma inhibin B, FSH, LH or total testosterone was observed between two TESEs in the control group (Additional file 1: Figure S1).

### Therapeutic response in the testes

**Fig. 5:**
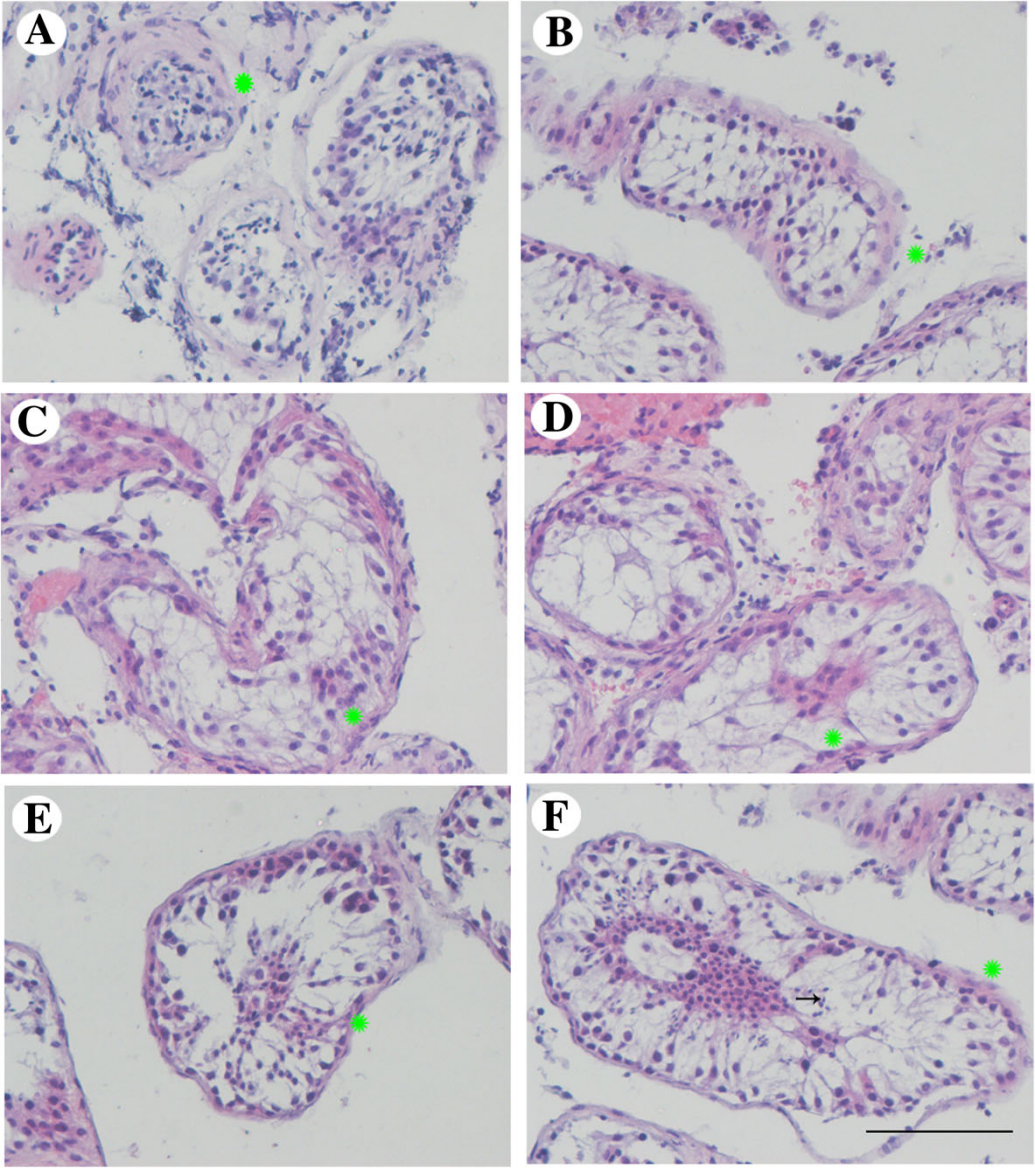
H&E staining of the testes before and after treatment. **a** The typical image of the testes specimens from OA patients. **b** The typical image in the first TESE. **c**-**f** The typical image of No response group, Response group 1, Response group 2A, Response 2B during the secondary TESE. Scale bar, 100 μm. Green color represented the Sertoli cells. The arrow indicated the mature sperm

**Fig. 6:**
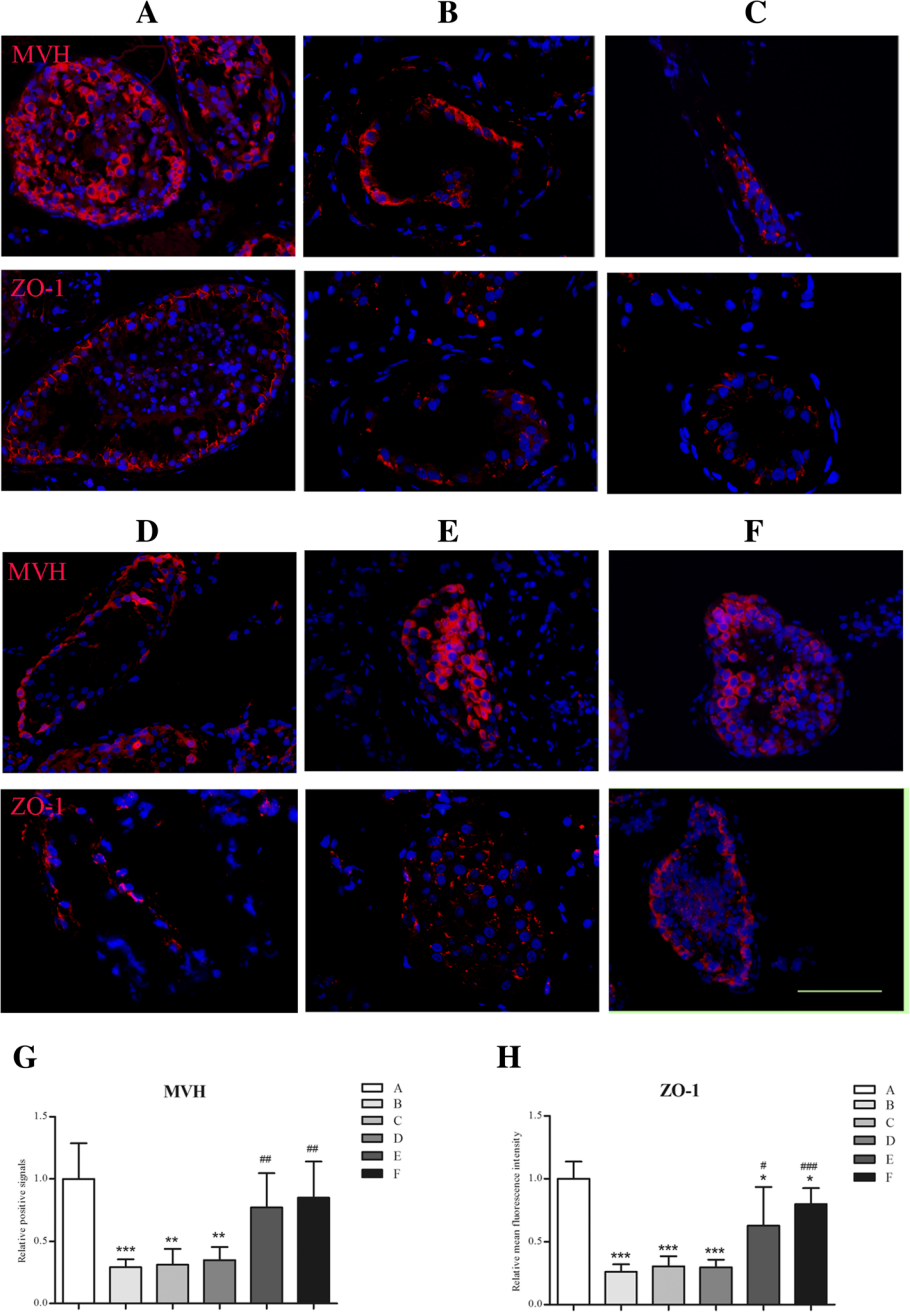
Immunofluorescence staining MVH and ZO-1 in the testis specimens. **a** The OA specimen were selected as positive control. **b** The typical images in the first TESE. **c**-**f** The typical images of patients in No response group, Response group 1, Response group 2A, Response group 2B during the secondary TESE. Scale bar, 100 μm. **g** Quantification of MVH-positive cells per tubule area in each group was then counted, 30 tubules from each group were calculated. **h** Relative mean fluorescence intensity of ZO-1 was calculated as total fluorescence intensity per tubule area, 30 tubules from each group were calculated. Results were shown as mean + SD. *P < 0.05, **P < 0.01, ***P < 0.001 compared with OA group; ^#^*P* < 0.05, ^##^*P* < 0.01, ^###^*P* < 0.001 compared with NOA patients during the first TESE.

H&E staining of the testis specimens were performed. No significant difference was found in the No response group (Fig. 5c) or Response group 1 (Fig. 5d) between the two TESEs. Germ cells in the Response group 2A were increased significantly but no mature spermatozoa were found (Fig. 5e). Excitingly, mature spermatozoa were observed in the Response group 2B (Fig. 5f). No obvious morphological change was found in Sertoli cells. Furthermore, MVH and ZO-1, markers of germ cells and BTB, were stained in the testis specimens. As shown in Fig. 6b, positive MVH signals were rarely observed in the seminiferous tubules and the expression and distribution of ZO-1 was abnormal (punctuate and discrete) in the NOA patients compared with OA patients whose ZO-1 distribution was consecutive (Fig. 6a). During the secondary TESE, the expression of MVH and ZO-1 were slightly affected in both No response group (Fig. 6c) and Response group 1 (Fig. 6d). However, the positive MVH signals and expression of ZO-1 were significantly increased in the Response group 2A (Fig. 6e), but the distribution of ZO-1 was not changed. Interestingly, not only mature spermatozoa were observed but also the expression and distribution of ZO-1 were recovered in a degree in the Response group 2B (Fig. 6f). Moreover, the MVH-positive cells and mean fluorescence intensity of ZO-1 per tubule area were then quantified as shown in Fig. 6g and h.

### Side effects

The treatment was well tolerated by all the enrolled patients and no cases offended with side effects. During GnRHα treatment alone in the first 4 weeks, 40% of the patients exhibited symptoms of androgenic deprivation such as mild loss of libido, erectile dysfunction, asthenia etc. hCG therapy in the follow-up treatment restored the concentration of testosterone and abolished these side effects. During the whole period, no significant difference of BMI, blood pressure or testicular volume was observed. Gynecomastia was not seen in any of the subjects. Hematological parameters including blood corpuscle, biochemical and lipid parameters remained stable during the whole treatment period (data not shown). No other side effects were discovered in the enrolled patients.

## Discussion

As mentioned previously, TESE or micro-TESE combined with ICSI was the only recommended treatment for NOA patients till date [1, 2]. However, the patients who failed to acquire sperm in the TESE remained a problem in the whole world. A series of studies have been reported regarding the treatment strategies for NOA patients including empirical medical therapies like testolactone [27, 28], hormone replacement therapy etc. [8, 9]. However, the results were not ideal. In 2012, Shiraishi and coworkers tried to trigger ‘gonadotropin reset’ with high-dose hCG stimulation. As a result, the high gonadotropins in NOA patients were decreased and the ability of testicular spermatogenesis was improved [7]. This indicated that gonadotropin reset is an efficient approach in the NOA patients. However, the dose of hCG was too high in clinical practice. Therefore, we enrolled patients with NOA and explored another way to fulfill gonadotropin reset with GnRHα in the present study.

As we all know, spermatogenesis is regulated via a complex array of paracrine, endocrine and juxtacrine regulatory cross-talk involving Leydig, Sertoli, peritubular, germ cells etc. [29]. FSH and testosterone were believed to play vital roles in spermatogenesis by stimulating Sertoli cells [30]. However, the precise mechanism still remain unclear. Studies in animals demonstrated that FSH was involved in the proliferation of early stage germ cells, and testosterone is one of the most important factors for initiating and maintaining spermatogenesis [31, 32]. As we all know, plasma FSH levels were often elevated in patients with NOA [7]. Significantly elevated FSH levels had been demonstrated to decrease FSH-FSHR signaling pathway in the testes of NOA patients. The possible explanation may be: (i) the uncoupling reaction of the FSHR from the effector system led to the constant phosphorylation of the C-terminal, intracellular domain of FSHR [33, 34]; (ii) decreased number of FSHRs was mediated by extensive clustering and internalization of the FSH–FSHR complex and by reduced expression of the receptor as a result of both decreased transcription levels and reduced half-life of mRNA [15, 35]. In view of the effects of high endogenous FSH, Foresta and coworkers have demonstrated that, leuprolide acetate treatment decreased the high endogenous gonadotropin levels, thus activating the FSH receptors. Followed by stimulation with exogenous FSH, the semen quality of the patients were significantly improved [11]. Based on these results, we tried to regulate endogenous gonadotropin levels with goserelin in the NOA patients in the present study. As a result, inhibin B was significantly lower than normal range in the NOA patients (Table 1), indicating injuried function of Sertoli cells. Interestingly, inhibin B levels were elevated in 11 of the 25 patients, indicating Sertoli cell function was improved after inhibition of endogenous gonadotropins in the first 4 weeks. The other 14 showed no response. This may be due to the excessive damage of Sertoli cells in the testis as the basic plasma inhibin B levels were significantly lower than the Response group (*p* = 0.009, Fig. 4f) which is consistent with the result of Foresta and coworkers who had demonstrated that the function of Sertoli cells was severely injuried and remained irresponsive to hormone treatment while the basic plasma inhibin B was low [11]. During the following 20 weeks, only 5 patients of the Response group (Response group 2) showed a constant increase of inhibin B, while the other 6 (Response group 1) did not. There was no difference observed in the basic plasma inhibin B levels between Response group 1 and 2, but an increased tendency was shown in Response group 2 (*P = 0.06*). This might possibly be due to that the function of Sertoli cells were incomplete in the Response group 1, i.e., the cells were not enough to initiate and maintain spermatogenesis as there was no significant change of the MVH signals after treatment (Fig. 5d and 6d). The inhibin B levels would be higher in presence of germ cells as they are also considered to be the source and involved in the secretion of inhibin B [36, 37]. Correspondingly, significant increase of positive signals of MVH in Response group 2 indicated the proliferation and meiosis of the germ cells after the treatment (Fig. 6e‑h), therefore, the inhibin B levels were elevated. All of these indicated that plasma inhibin B level may act as a good marker to predict spermatogenesis in the testis and to evaluate the response of the therapies in the NOA patients.

As mentioned previously, BTB played an important role in the spermatogenesis [19]. Aberrant expressions of TJs were associated with abnormal spermatogenesis [38]. ZO-1, occludin, and claudin are the TJ proteins identified in the testis [39]. In the present study, the distribution of ZO-1 was punctuate and discrete in NOA patients as shown in Fig. 6b compared with patients of OA whose ZO-1 distribution were normal and consecutive. The expression and distribution of ZO-1 were improved in Response group 2B with mature spermatozoa were observed in the testis while only expression of ZO-1 was improved in Response group 2A with no mature spermatozoa, although the number of germ cells was increased significantly (Fig. 6e, f). This indicated that well-distributed ZO-1 played an important role in the sperm maturation changes, which was consistent with other studies [39, 40]. Under the physiological conditions, the main factor affecting the TJ formation is the endogenous testosterone [29]. Besides, FSH may also play a key role in the expression and localization of TJ proteins [29]. The ameliorative expression and distribution of ZO-1 in the Response group 2B also indicated the effectiveness of the protocol in restoring the function of FSHRs and LHRs and the elevation of intra-testicular testosterone levels by subsequent hCG treatment that has been demonstrated by others [9, 10].

As known previously, HP, MA and SCOS were the three main histological classifications of NOA [3]. SCOS is excluded here as no tubules contain germ cells in the testis specimens of the patients [41], hence the possibility of acquiring sperm was very low [6]. Spermatogenesis of MA was often arrested at the spermatogonial or primary spermatocyte stage. It was mainly related to the presence of a genetic lesion or toxicant exposure [41]. The process of spermatogenesis in HP was almost intact, but reduced to a certain extent. Hence, we supposed that it was related with terrible testis microenvironment and improve the microenvironment via hormone may be beneficial for spermatogenesis [7]. HP were also believed to be the ideal objects for medical treatment in the previous studies [6, 7]. Moreover, the cost of the treatment was high. Therefore, the clinical trial was performed in patients who are more likely to succeed. Besides, there is a deficiency in the present study. The effectiveness of the protocol in the study was lack of strict “control group” who should be given only hCG combined with hMG in the process, that is to say, the role of goserelin in the study was lack of evidence. However, the elevation of inhibin B in the first 4 weeks during which goserelin was given alone indicated the positive effect of it. Moreover, Foresta and coworkers had demonstrated that FSH alone would not enhance plasma inhibin B in patients with high endogenous gonadotropins as the Sertoli cells were irresponsive [11]. Therefore, the reactivity to FSH was restored via the use of goserelin in the present study as reflected by constant increased inhibin B following hMG treatment was another proof for the role of goserelin, which is also consistent with the result of Foresta and coworkers [11].

## Conclusions

This was a preliminary prospective study, and we initially explored the therapeutic protocol in patients with NOA. Although only 2 of the 25 treated patients succeeded to acquire sperm, the plasma inhibin B levels were significantly elevated in 5 patients (25%). This indicated the effectiveness of the protocol and plasma inhibin B may be a good biomarker in the prediction of spermatogenesis. Destruction of BTB may be related with the arrest of spermatogenesis in the NOA patients, while ameliorative BTB would be beneficial for spermatogenesis, indicating that BTB might be a therapeutic target in the NOA patients. In a word, ‘gonadotropin reset’ was able to improve the ability of testicular spermatogenesis in the NOA patients which may be a result of restored sensitivity of Sertoli and Leydig cells to gonadotropins. More rigorous randomized controlled trial studies should be carried out to explore the effect of the therapeutic protocol in NOA patients. The limited success rate of the present study indicated us that the etiology or mechanism of NOA should be further studied and more efficient therapeutic protocols should be explored.

## Additional file

Additional file 1: **Figure S1.** The change of plasma hormones in the control group. The comparison of plasma FSH **(A)**, LH **(B)**, Testosterone **(C)**, inhibin B **(D)** between two TESEs. Results were shown as mean + SD. *p* > 0.05 in all the comparisons. (TIF 316 kb)

## Abbreviations

BTB: Blood tesis barrier
FNAC: Fine needle aspiration cytology
GnRHα: Gonadotropin releasing hormone agonist
hCG: Human chorionic gonadotropin
hMG: Human menopausal gonadotropin
HP: Hypospermatogenesis
ICSI: Intracytoplasmic sperm injection
MA: Maturation arrest
NOA: Non-obstructive azoospermia
SCOS: Sertoli cell only syndrome
TESE: Testicular sperm extraction
ZO-1: Zonula occludens-1
