# Supplementary figures and images for: Spermatogenesis improved by suppressing the high level of endogenous gonadotropins in idiopathic non-obstructive azoospermia: a case control pilot study
Source: Reprod Biol Endocrinol. 2018 Sep 22;16:91. doi: 10.1186/s12958-018-0401-7 (PMC6150963; doi:10.1186/s12958-018-0401-7)

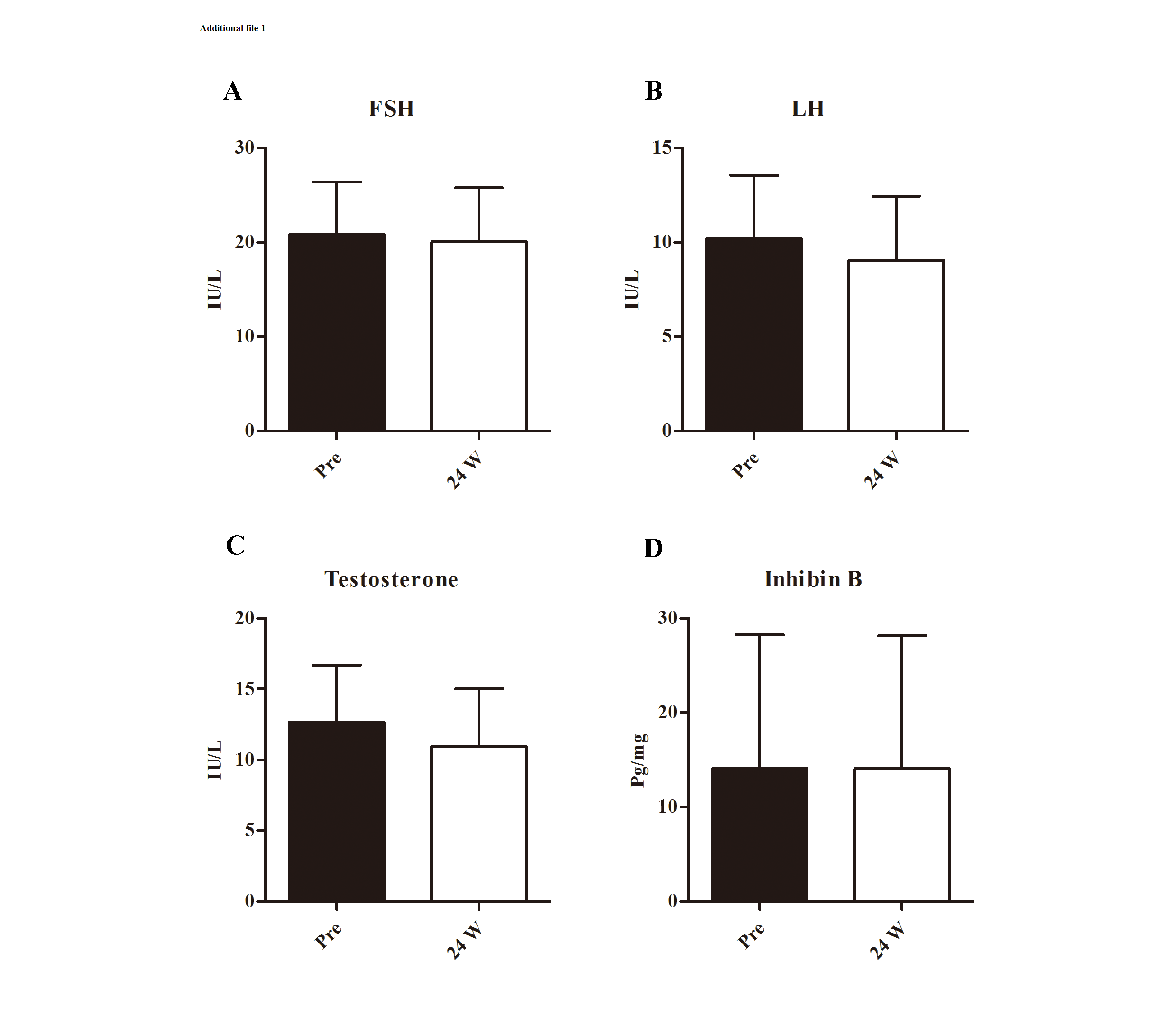

Supplement: Supplementary file 1 — Figure S1. The change of plasma hormones in the control group. The comparison of plasma FSH (A), LH (B), Testosterone (C), inhibin B (D) between two TESEs. Results were shown as mean + SD. p > 0.05 in all the comparisons. (TIF 316 kb) [file 12958_2018_401_MOESM1_ESM.tif]
